# NOVEL MODALITIES FOR UNCOVERING BRAIN MECHANISMS UNDERLYING COGNITIVE AND MOTOR FUNCTIONS

**DOI:** 10.1093/geroni/igae098.1055

**Published:** 2024-12-31

**Authors:** Sarah Fraser, Andrea Rosso, Teresa Liu-Ambrose

**Affiliations:** Chair; Co-Chair; Discussant

## Abstract

It is now well-established that age-related declines in cognitive and motor functions co-occur and that shared neural resources are involved. However, details of these neural resources are still largely unknown, limiting development of effective prevention and intervention strategies. This symposium aims to answer: What can neuroimaging tell us about subjective cognitive decline and what changes in gait and mitochondrial function are associated with changes in the brain? How does dopamine alter these associations and help promote resilience in the brain? A series of recent studies using neuroimaging technologies aimed to answer these questions and provide new information on neural resources: (1) Salzman et al. will share their work using functional near infra-red spectroscopy (fNIRS) and dual-task tapping to explore cerebral oxygenation differences in older adults with and without subjective cognitive decline; (2) Rosso et al. explored if neural inefficiency or compensation measured by fNIRS during dual-task walking was influenced by dopamine binding using Positron Emission Tomography (PET); (3) Rosano et al. employed PET to explore associations between dopaminergic binding and gait speed changes when transitioning to an uneven surface; and finally (4) Tian et al. examined the relationship between skeletal muscle mitochondrial function via MR Spectroscopy and longitudinal brain structural changes via magnetic resonance imaging (MRI) and DTI data from the Baltimore Longitudinal Study on Aging. Each of these studies contributes to the evidence base on neuroimaging correlates of cognitive and motor function and helps us to understand what factors foster resilience and maintenance of these functions with age.

